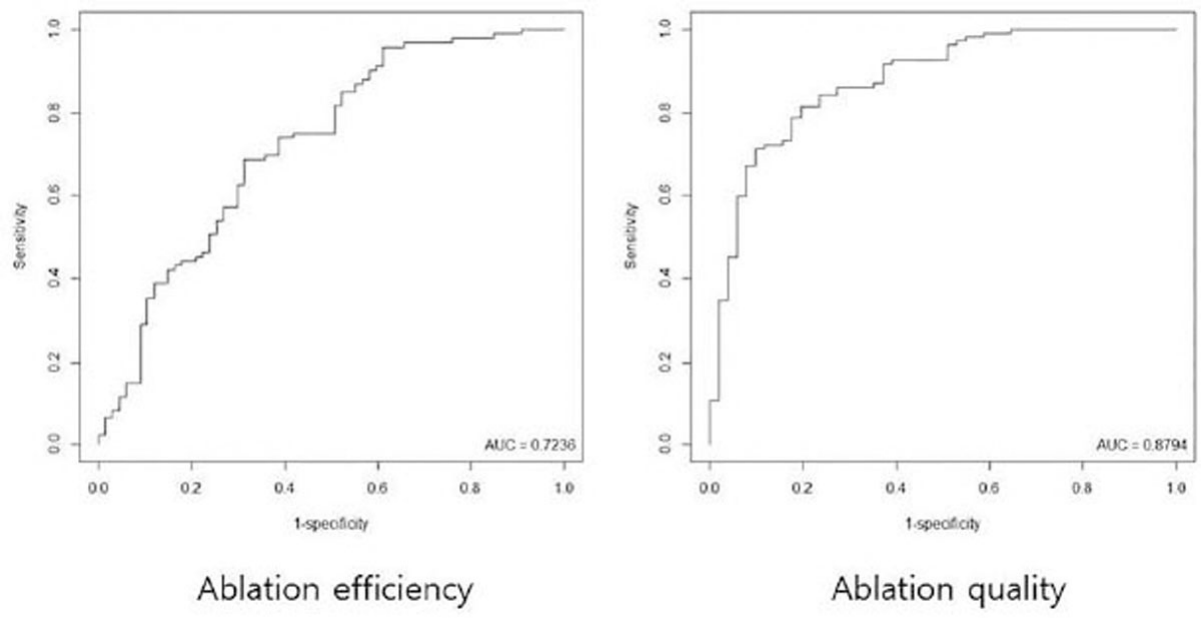# Screening MRI-based prediction model for therapeutic response of MR-HIFU ablation of uterine fibroids

**DOI:** 10.1186/2050-5736-3-S1-O98

**Published:** 2015-06-30

**Authors:** Young-sun Kim, Bilgin Keserci, Hyo Keun Lim, Hyunchul Rhim

**Affiliations:** 1 Samsung Medical Center, Seoul, Republic of Korea; 2 Philips Healthcare, Seoul, Republic of Korea

## Background/introduction

With regards to MR-HIFU ablation of uterine fibroids, there have been no screening MR criteria that comprehensively consider multiple influencing factors. The aims of this study was to generate screening MRI-based prediction model for therapeutic responses of MR-guided high-intensity focused ultrasound (MR-HIFU) ablation of uterine fibroids comprehensively considering multiple influencing factors.

## Methods

A total of 160 symptomatic uterine fibroids (diameter 8.3cm, range 3.1-15.0cm) in 112 women (age 43.3, range 25-55) who were treated with MR-HIFU ablation were retrospectively analyzed. The following three parameters of screening MRI were evaluated. 1) Subcutaneous fat was measured as a thickness of the most compressed point (mm) on prone position. 2) Relative peak enhancement (%) was calculated based on time-signal intensity curve analysis of fibroid in perfusion MRI (100 dynamics, 3s time resolution), in which 0% refers the same signal intensity as in precontrast image. 3) Signal intensity was assessed as a ratio of T2 signal intensity of uterine fibroids to that of skeletal muscle. Those parameters were used to generate prediction models with regards to ablation efficiency (i.e., non-perfused volume/treatment cell volume) and ablation quality (grade 1~5, from poor to excellent), respectively, using generalized estimating equation (GEE) analysis. Then, cut-off values for successful treatment (ablation efficiency >1.0; ablation quality grade 4 or 5) were determined based on receiver operating characteristic (ROC) curve analyses.

## Results and conclusions

GEE analyses produced the models of “y1=2.2637– 0.0415x1–0.0011x2–0.0772x3” and “y2=6.8148–0.1070x1–0.0050x2–0.2163x3”, where y1=ablation efficiency, y2=ablation quality, x1=subcutaneous fat thickness, x2=relative peak enhancement, and x3=T2 signal intensity ratio (p-values for x1, 0.0068 and <0.0001; for x2, 0.1952 and 0.0001; for x3, <0.0001 and <0.0001, respectively). Cut-off values for successful treatments based on ROC curve analyses turned out to be 1.312 for of ablation efficiency (AUC, .7236; sensitivity, .6882; specificity, .6866) and 4.019 for ablation quality (AUC, .8794; sensitivity, .7156; specificity, .9020).

Conclusion: Simple equation models to predict therapeutic responses of MR-HIFU ablation of uterine fibroids in terms of ablation efficiency and quality were generated, which are easily applicable to screening MRI.

**Figure:**